# Dictionary of General and Ophthalmic Surgery

**Published:** 1839-10

**Authors:** 


### Art. VII.

1. *Handwörterbuch der gesammten Chirurgie und Augenheilkunde.* Herausgegeben von Ernst Blasius, Professor der Chirurgie an der Universität zu Halle, &c. Erste Band. Erste Hälfte. — *Berlin,* 1836. 8vo.

*Dictionary of General and Ophthalmic Surgery.* Edited by Dr. Ernest Blasius, Professor of Surgery at Halle, &c. Vol. I. Part I.— *Berlin,* 1836. (Art. *Aneurism.*)

2. *The Cyclopædia of Practical Surgery.* Edited by William B. Costello, m.d. Parts II. and III.—*London; August,* 1837, and *July,* 1838. 8vo. (Art. *Aneurism,* by James Wardrop, m.d.)

In taking up the last part (Pt. III.) of Dr. Costello's Cyclopædia, for the purpose of noticing the article on Aneurism, by Dr. Wardrop, we could not but think that its editor and publishers must be blessed with a more than ordinary share of that stoical philosophy which sets at defiance all the thunders of the press. Each number of this work, as some of our readers know to their cost, and as others will be amused to hear, professes to follow its immediate predecessor after an interval of two months, "Published in Parts every alternate month." Yet so little has this announcement been fulfilled, that "every alternate year" would be nearer the truth. In fact, judging from the past progress of the work, we think the term *Century* might properly supersede that of *Cyclopædia,* since, by a moderate computation, not less than an hundred years will elapse before it can be completed. That its editor and contributors may live to see this desirable consummation, we earnestly hope; but as we ourselves cannot, in the ordinary course of events, expect to be in at the death, we must be content to catch, as we can, its juvenile numbers, leaving its maturer pages to the consideration of our successors, to the third or fourth generation, in the editorial chair.

It is not our purpose here to enter into an investigation of the various modes in which aneurism may be formed; these are to be found detailed in most elementary works, also in numerous treatises more expressly devoted to the consideration of this subject. On the present occasion we are rather desirous of illustrating the great practical principles which must be kept in view in the attempt to remedy the mischief occasioned or threatened by an aneurismal dilatation, and to show how far the measures recommended are calculated to effect the proposed object. The cure of aneurism may be effected spontaneously, that is, by certain changes brought about in the local condition of the affected vessel, without the assistance of the physician or surgeon; by certain methods of constitutional or internal treatment; and by local surgical operations. The one great principle, however, upon which all these modes of effecting a cure act, whether by the unassisted operations of the system or through the intervention of art, is the inducing of such a degree of remora in the local circulation as shall lead to the ultimate formation of a coagulum in the dilated portion of the diseased artery, thereby reducing the caliber, or producing complete obliteration of the affected vessel.

Dr. Wardrop says that the spontaneous cure of aneurism may take place in no less than five different ways.

"1. The first and the most common of these spontaneous processes of cure is effected by the aneurismal sac being so filled up and strengthened with concrete fibrine that all danger of a rupture of the sac is removed, whilst, at the same time, the original canal of the vessel remains pervious, and carries on the circulation of the blood. 2. In the second process, by which an aneurism may undergo a spontaneous cure, the sac is not only filled with concreted fibrine, but the canal of the artery is obliterated. 3. An aneurism may likewise undergo a spontaneous process of cure by the tumour acquiring such a size and position that, by its pressure on the trunk of the artery, either on the cardiac or on the capillary side of the tumour, the sides of the artery are brought into contact and adhere. 4. An aneurism may also be cured spontaneously by a process of suppuration in the sac, and the artery above and below the tumour having previously been filled with coagulum. In such a case the integuments inflame and adhere to the sac, and then ulcerate, and those portions of the coagulum which cannot be removed by the process of absorption are ejected through the ulcerated opening, allowing the cavity of the sac to be filled up and obliterated by the process of granulation. 5. The fifth mode of spontaneous cure has been observed from the bursting of the tumour underneath the common integument, and the artery becoming obliterated by the pressure of the effused blood." (p. 206.)

It cannot be doubted that four out of the five methods here enumerated are merely illustrations of the principle which we have stated above. The first, however, and most common is not exactly parallel to these, and accordingly Dr. Wardrop would seem, with respect to this individual process, to have felt some difficulty as to including it within a general law, applicable to all the other processes of cure, whether spontaneous or artificial, with which we have hitherto become acquainted. "The principle or [of?] the processes which Nature employs for the cure of the disease," he observes, "is in all of them the same, the parietes of the sac being strengthened by, or the whole cavity filled with a fibrinous concretion." This *strengthening* of the parietes of the sac by fibrinous concretion we hold to be a mere assumption, fitted to meet a presumed difficulty, and of no value whatever in the true rationale of the process. It is true that Dr. Wardrop subsequently admits that this "fibrinous concretion is caused, or is permitted to be formed, whenever the circulation of the blood within the sac becomes preternaturally languid;" but by taking up with the intermediate condition of a presumed strengthening of the walls of the sac, he has obscured the expression of the leading principle, and introduced an element into his reasoning which a closer examination will show to be incorrect.

It is probable that Dr. Wardrop, in making this statement, may have been influenced by his peculiar views as to the formation of this concretion, the precise nature of which, he says, has not been accurately pointed out by pathological enquirers.

"When the fibrinous concretion is examined," he observes, "it is found to consist of numerous concentric laminæ, which are more or less easily separable from one another and firmer in proportion as they approach nearer to the coats of the sac. The laminæ of fibrine which are in immediate contact with the blood circulating in the sac have generally a flocculent appearance, and have coagula of red blood mixed with them. But in some aneurismal tumours the concreted fibrine has its interior surface smooth and polished, being lined throughout by a membrane which has the appearance, as already mentioned, of being continuous with the lining membrane of the artery. . . . . Though this fibrinous concretion can be easily separated from the sac, yet it adheres closely to its internal surface; and I have every reason to believe that there is a vascular connexion existing between them. . . . . There is evidently a great difference, however, in the anatomical characters of a common clot of blood and a fibrinous concretion; and the coagulated lymph or fibrine, which is deposited in an aneurism appears to me to bear a strong analogy to the internal clot found in an artery on which a ligature has been placed, and likewise with those polypiform concretions which are formed within the cavities of the heart. Besides the coagulum of blood which is formed within the canal of an artery, immediately after the application of a ligature, and which acts merely as a temporary barrier to the flow of the blood, there is subsequently an exudation of fibrine from the internal coat of the artery, commencing from the place of the ligature, and extending as far up the canal as that point from which the first branch is sent off from the trunk of the artery. This fibrine adheres intimately, and appears to have a vascular connexion with the internal coat of the vessel. . . . . That the fibrinous concretions found within an aneurismal sac are organized also appears extremely probable, from the masses of coagulable lymph which are found within the cavities of the heart, in many instances adhering to, and having a distinct vascular connexion with, the endocardium or lining membrane of the heart; but what appears to me to confirm the opinion of the fibrinous concretion in an aneurismal sac being organized, and being an effusion from the vasa vasorum, is the fact of the coagulum formed in an obstructed vein having been distinctly injected by Mr. Kiernan. A branch of the vena porta had been compressed by a tumour and plugged up by effused lymph, and the arteries having been filled with fine injection, the coagulum was reddened at some points, and a very considerable sized vessel is exhibited in the preparation passing from the internal coat of the vein into the coagulum. Whilst, therefore, red blood may coagulate in an aneurismal tumour, merely by the diminution in the force of the circulation, as shall hereafter be pointed out, the formation of an organized fibrinous concretion must be the result of a different process, and can only be explained by supposing fibrine to be effused from the internal surface of the sac itself, in like manner as coagulable lymph is effused on the surface of an inflamed serous membrane." (pp. 208, 209.)

We have no objection to the adoption of Dr. Wardrop's views, could we see but the shadow of a reason brought forward in proof of their correctness; but without taking into consideration the inapplicability of Mr. Kiernan's preparation, which is the only fact adduced in support of them, it may at once be said that, had these views been founded in what actually occurs, the author would have been at no loss to have confirmed them by instances passing daily under his own observation and that of his professional friends. The distinction of the contents of an aneurismal sac into layers of fibrinous concretion and mere coagula we believe to be based on correct observation; but the theory of the formation of these concretions and the idea that they contribute in any appreciable degree to the strengthening of the parietes of the sac are not only unsupported by facts, but also, as it appears to us, of a very questionable nature.

In such of the larger aneurismal swellings as we have had an opportunity of examining after death, the layers of fibrinous concretion have been of no firmer consistence than so many pieces of well-soaked brown paper, and have appeared thoroughly macerated, as it were; and, so far from presenting any impediment to the impulse of the heart's action, were easily lacerable, yielding readily to the application of the slightest force. The preparation of Mr. Kiernan, we have said, is inapplicable; for, independently of the fibrinous deposit in this instance having occurred under entirely dissimilar circumstances, the subject of vascular connexions existing between lymphy exudations and the parts with which they are in immediate contact is involved in too much obscurity to found any very definite conclusions upon. The fibrinous exudation which takes place from the internal coat of the artery subsequent to ligature is also by no means a parallel to what occurs in an aneurismal sac. In the preparation in the Hunterian Museum, referred to by Dr. Wardrop, in which the internal clot was reddened by the injection of the vasa vasorum of an artery to which a ligature had been applied, John Hunter was himself doubtful whether the red colour of the clot was owing to injected vessels or the effect of effusion. But, admitting that this preparation proves the fact of vascular connexion in the class of instances to which it especially refers, it can scarcely be considered that more than a very remote analogy exists between the state of an artery rendered impervious by ligature and rupture of the internal coat, and aneurismal dilatations in which the inner coat of the vessel is often preserved intact and the passage remains free.

We must, however, be contented with a mere indication of these objections, and proceed to the consideration of the internal and external methods of cure adopted in the treatment of aneurism. It is unnecessary to enter at any length into details upon this point, and it will be sufficient to state that the principle before laid down is that upon which all of them are founded. The chief means employed in the internal mode of treatment are the hunger-cure of Valsalva, by which it is attempted so to reduce the force and lessen the amount of the circulating fluid as to favour the formation of a coagulum in the dilated portion of the vessel. To this the repeated abstraction of blood and the exhibition of digitalis, hydrocyanic acid, and tartarized antimony, materially contribute; and there can be no doubt that many cases of aneurism have yielded to the steady and well-directed employment of these measures.

The external and local methods of compression and ligature of the diseased vessel above the aneurismal swelling are obviously mechanical measures, at least in their primary operation, for the attainment of the same object. The various modes of applying compression are well pointed out and described by Dr. Blasius ; but, besides being inapplicable to a vast number of instances, compression is very uncertain in its effects and tedious in operation in many of those in which it promises a favorable result. The operation by ligature, then, is that which is chiefly trusted to in the present day ; and of this several modifications have been devised. The method at one time followed was to lay open the aneurismal swelling, turn out the contents of the sac, and place a ligature on the artery, above and below the dilated part, the cavity becoming gradually filled up through the suppurative and granulating processes.

This mode of operating, however, is now rarely if at all had recourse to in this country, although, from the observations of Dr. Blasius, it would still seem to be occasionally practised in Germany in certain forms of diffuse aneurism and of aneurism by anastomosis. That which is now most generally followed, as being the most simple and certain in its operation, and of the most general application, is the Hunterian operation, in which, as our readers well know, a ligature is placed on the arterial trunk, between the aneurism and the heart. The rationale of this operation and the subsequent progress, together with the measures requisite to ensure success, are now too generally known and appreciated to render it necessary to allude to it at any greater length.

Another method of operation by ligature was proposed by Brasdor, which consists in the application of the ligature beyond or on the capillary side of the tumour, and is considered by some surgeons of eminence to be applicable in certain cases, in which, from want of room to apply the ligature on the cardiac side of the aneurism, the Hunterian operation is impracticable. Brasdor's operation was never performed by himself, and two cases, the one of femoral and the other of iliac aneurism, operated upon respectively by Deschamps and Sir Astley Cooper, after this method, having terminated fatally, it was not again tried until the year 1825, when Dr. Wardrop practised it in a case of aneurism of the right carotid artery. This case was published in the Transactions of the Medico-Chirurgical Society for that year; and its successful result led to the adoption of the practice in other instances. One of the results of this method of operating, and which could scarcely have been anticipated, is the immediate diminution of the size of the tumour. This diminution in Dr. Wardrop's case was progressive, "so that on the fourth day after the operation it (the tumour) seemed to have diminished nearly one third, the upper and tracheal portion had lost all pulsation, and only the scapular portion retained an obscure undulatory thrill."

A modification of this operation has been since devised by Dr. Wardrop, and successfully put in practice by himself and other eminent surgeons. An objection to Brasdor's operation was stated by Mr. Hodgson, in alluding to the unsuccessful case of Deschamps: "If a stream," he observes, "continued to pass through the aneurism into branches which originated below it, the blood contained in the tumour was *not at rest*, and consequently did not coagulate; a cure *could not*, therefore, be expected to ensue on the principle which led to the performance of the operation." And again, "It must be acknowledged, however, that it will be almost impossible in any case to ascertain that *no branch* arises from the sac, which, by continuing the circulation through the latter, may defeat the objects of the operation." The failure in this reasoning arises from its not having been recognized—what, however, might have been inferred from the success of many of the cases treated upon Valsalva's method—that the real principle of treatment is the inducing of a certain degree of diminution in the force of the circulation rather than its immediate and entire stoppage.

Dr. Wardrop, in quoting Mr. Hodgson's remarks, observes, "From the important pathological fact that in the processes which are employed by Nature for effecting the 'spontaneous cure' of an aneurism, and likewise from the circumstance that in some cases of popliteal aneurism, where the Hunterian operation had been performed, a complete stoppage of the circulation of the blood within the tumour had not been required, it appeared to me a legitimate conclusion that the progress of an aneurism might be arrested, or even the tumour consolidated, by placing a ligature *on one of the branches* of a diseased trunk. It being admitted that a diminution of the force of the circulation of the blood, through an aneurismal sac, was sufficient to cure the disease, the important question still remained to be determined—to what degree is it required to diminish the momentum of the circulation through an aneurism, in order to permit a fibrinous concretion to be formed within the sac?" The importance of keeping in view the distinction between mere coagulum of red blood and layers of concrete fibrine is then insisted upon; and it is attempted to be shown that while the operations of Hunter and Brasdor are contrived upon the principle of putting a complete stop to the circulation in the tumour, the modification of the latter of these, introduced by the author, is more on the principle of the spontaneous cure.

As it appears to us, however, setting aside the questionable opinion of a strengthening of the parietes of the dilated artery by these layers of fibrinous concretion, the same principle includes both the spontaneous cure, the method of Valsalva, and the operation of Wardrop, on the one hand, and the methods of Hunter and Brasdor on the other: the check to the circulation in these last, as also in some of the instances of spontaneous cure, being complete instead of partial. In aneurisms of the lesser arterial trunks the disadvantages attending the carrying out of the principle to its *extreme*, by putting an immediate arrest on the course of the circulation, though occasionally felt, are still of trifling import, compared with the greater certitude attending the operation; but in the dilatations of the larger vessels, of the carotids and subclavians near their origin from the arteria innominata and arch of the aorta, and in aneurism of the aorta itself, the case is widely different. Much credit is due, therefore, to Dr. Wardrop, for the devising and carrying into operation the method which he has proposed; and the success which, in a certain number of instances at least, has attended it affords encouragement for the following out of the principle, whether by operation or by other modes of treatment, in all cases in which the Hunterian method or that of Brasdor is, from whatever cause, inapplicable. Dr. Wardrop relates six cases of aneurism of the arteria innominata, operated upon after the mode which he advocates: the first by himself, that of Mrs. Denmark, already before the public; the second by Mr. Evans, of Belper; the third by Professor Mott, of New York; the fourth by Dr. Morrison, of Buenos Ayres; the fifth by M. Laugier; the sixth by Mr. Fearn, of Derby—all treated by placing a ligature *on one of the branches* of the diseased artery. He refers also to two others, in which a similar principle of operating was adopted, one by Mr. Scott and the other by Mr. Key. The results of these operations are thus commented upon:

"In five of the cases the operation was followed by relief of all the symptoms, and by a diminution of the bulk and consolidation of the tumour. The three other cases were unsuccessful; but this militates not against the principle of the operation itself, but the injudicious selection of the patients on whom it had been employed. In M. Laugier's patient the consecutive hemorrhage was a proof of the diseased state of the artery at the place where the ligature was applied; and the appearances after death pointed out that the operation, in this instance, where the aneurismal disease was so extensive, was by no means judicious. Neither can the unsuccessful result of the case operated upon by Mr. Key, where the patient died a few hours after the operation, be considered as any proof of the disadvantages of this mode of operating, but of its misapplication; and in the case which was operated upon by Mr. Scott there is every reason to believe that the aneurism was too far advanced, and the vessels too extensively diseased to admit of any benefit from the operation." (p. 236.)

With this extract we must conclude our notice of Dr. Wardrop's able article, regretting that we are unable, on account of their length, to transfer any one of the cases to our pages. For a further development of the views of the author, and for the consecutive advantages of his mode of operating in the instances of aneurism of the primary branches of the arterial system, we refer to the essay itself. The article of Dr. Blasius, though inferior in some respects, is yet of equal if not superior utility for the purpose for which it is intended. It affords a succinct account of the nature, varieties, etiology, and development of aneurisms; of their symptoms, diagnosis, and progress, with a good summary of the several modes of treatment; and is calculated to be of especial assistance to the young surgeon, from the fulness with which the directions for facilitating the mere manual part of the operations are given.

---

